# A Dynamic Radiographic Imaging Study of Lumbar Intervertebral Disc Morphometry and Deformation *In Vivo*

**DOI:** 10.1038/s41598-019-51871-w

**Published:** 2019-10-29

**Authors:** Ryan M. Byrne, Ameet K. Aiyangar, Xudong Zhang

**Affiliations:** 1 Department of Mechanical Engineering and Materials Science, University of Pittsburgh, Pittsburgh, PA 15203 USA; 2 Department of Orthopaedic Surgery, University of Pittsburgh, Pittsburgh, PA 15203 USA; 3 Mechanical Systems Engineering, EMPA (Swiss Federal Laboratories for Materials Science and Technology), 8600 Duebendorf, Switzerland; 4 Department of Industrial & Systems Engineering, Texas A&M University, College Station, TX 77843 USA; 5 Department of Biomedical Engineering, Texas A&M University, College Station, TX 77843 USA; 6 Department of Mechanical Engineering, Texas A&M University, College Station, TX 77843 USA

**Keywords:** Musculoskeletal system, Biomedical engineering, Mechanical engineering

## Abstract

Intervertebral discs are important structural components of the spine but also are significant sources of morbidity, especially for the “low back” lumbar region. Mechanical damage to, or degeneration of, the lumbar discs can diminish their structural integrity and elicit debilitating low back pain. Advancement of reparative or regenerative means to treat damaged or degenerated discs is hindered by a lack of basic understanding of the disc load-deformation characteristics *in vivo*. The current study presents an *in vivo* analysis of the morphometry and deformation of lumbar (L2-S1) intervertebral discs in 10 healthy participants while performing a common lifting act, using novel dynamic radiographic imaging of the lumbar vertebral body motion. Data analyses show uniquely different (p < 0.05) characteristics in morphometry, normal and shear strain patterns of the L5S1 discs, while the rest of lumbar discs exhibit great similarity. In particular shear strains in L2-L5 discs exhibited stronger linear correlations (*R*^2^ ≥ 0.80) between strain changes and amount of lumbar flexion-extension motion compared to L5S1 (R^2^ ≤ 0.5). The study therefore advances the state of knowledge on *in vivo* mechanical responses of the lumbar intervertebral discs during functional tasks.

## Introduction

Intervertebral discs are critical structural components of the spine, comprising the softer, more compliant portion that transmits approximately 80–90% of its axial loads, while providing almost all the mobility^1–3^. Degenerative or trauma-related changes to the intervertebral discs in the lumbar spine can lead to loss of structural integrity and, more importantly, debilitating chronic low back pain (LBP)^4^—one of the most prevalent disorders and the leading cause of years lived with disability, as shown by a Global Burden of Disease Study^5^. Given the unclear etiology of degeneration-related LBP and lack of an accepted disease model, comprehensive treatment remains elusive^6^. For example, currently available surgical interventions such as lumbar fusion or artificial disc replacement might successfully mitigate pain symptoms when conservative treatment fails, but may not fully restore joint function^7–11^. Furthermore, iatrogenic factors lead to altered mechanical responses resulting in sub-optimal long-term outcomes^12,13^. Tissue engineering-based repair or replacement solutions to restore structural *and* functional capabilities, while retaining the capacity to remodel in response to external stimuli^14^, present a promising treatment approach^15^. However, a lack of well-defined biomechanical functional benchmarks or design parameters with respect to the *in vivo* load capacity as well as deformation patterns has hindered successful translation of these approaches into clinical reality^16^.

Although a multi-factorial conundrum, changes in the *in vivo* mechanical environment and the ensuing changes in biochemical environment within the discs have been accepted as separate but inter-related contributing factors to disc degeneration. Consequently, there is a growing interest in clarifying the mechanobiological links between the mechanotransduction, biochemical environment, and overall *in vivo* mechanical environment^6,17^. While *aberrant* mechanical loading has been determined to affect intervertebral disc cellular response in *ex vivo* experiments^18–22^, there is limited knowledge regarding the *in vivo* mechanical environment of the lumbar intervertebral disc – such as stress and strain patterns – during dynamic functional activities. Studies employing direct intra-discal measurement techniques have generated limited, precious data to allow characterization of the intra-discal pressure distribution in various static positions^23–25^ and even estimation of spinal loads therefrom^24^. Though insightful, a major limitation of these studies has been the inability to measure shear stresses and strains^17^, which are thought, in part, to drive the degenerative cascade in the intervertebral discs^26,27^. Moreover, highly invasive, needle-based disc puncture techniques are now discouraged^17^ due to the risk of instigating disc degeneration^28^, and our understanding of *in vivo* loading relies primarily on computational models employing inverse static and dynamic analyses^29–36^.

Three-dimensional (3D) skeletal kinematics of the lumbar vertebrae *in vivo* during a load-lifting motion were successfully measured by our group recently using dynamic stereo-radiographic imaging^37^. The skeletal kinematic data, combined with subject-specific 3D vertebral bone morphometric data acquired using computed tomography (CT), afford an unprecedented opportunity to study the lumbar intervertebral disc morphometry and deformation *in vivo*. Here, we report such a study of the disc morphometry at two discrete end (reference) positions and continuous disc deformation over the entire range of motion (ROM) at five consistently definable regions of the discs.

## Results

Results on disc morphometry, measured as normalized disc height (nDH) between adjacent endplates, and disc strains are presented in different ways to visualize their variations along one or more of the following dimensions: (1) across lumbar segmental levels; (2) over the entire surface or transverse planar area; (3) between two discrete positions, the flexed position at the beginning and the upright standing position at the end of a motion; (4) over time or the range of motion; and (5) across five selected, consistently identifiable disc regions: anterior, posterior, left, right, and center.

### Intervertebral disc height

**Figure 1:**
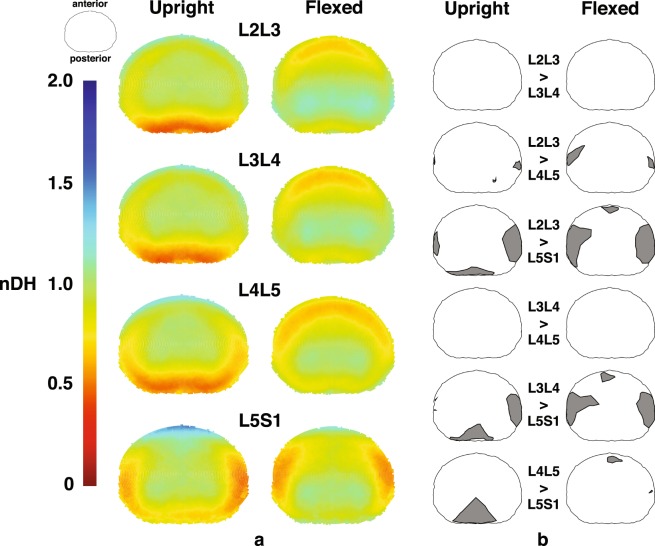
Mapping of nDH across the axial planar surfaces of the lumbar discs from the L2 to S1. (**a**) Distribution of nDH across the whole disc at the upright (left) and flexed (right) positions. Green (nDH = 1) represents the nDH at the disc center, while red and blue represent locations of small and large nDH, respectively, compared to the disc center. (**b**) Areas exhibiting significantly different nDH values between segment levels at the upright (left) and flexed (right) positions based on non-overlapping ±95% confidence intervals.

**Figure 2:**
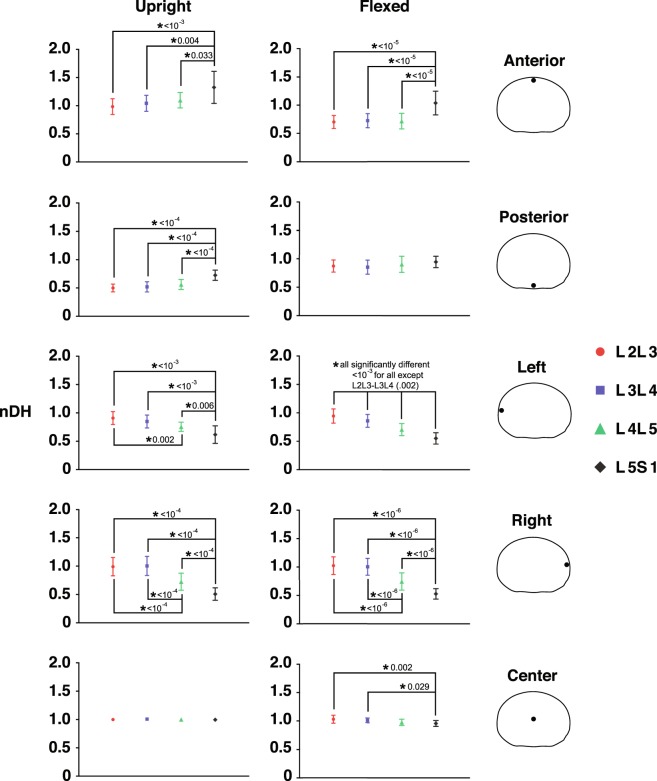
Average normalized disc height (nDH) of five regions of the disc at each segment level. nDH is calculated at the anterior, posterior, left, right, and center regions of the disc at the upright (left) and flexed (right) position. Error bars represent ±95% confidence intervals. An asterisk indicates a significant difference between segment levels and is accompanied by its associated p-value as assessed by Repeated Measures ANOVA.

The nDH measurements for trials of different external load magnitudes for each subject are pooled, as no load effect is observed across each disc’s entire transverse planar area. The L5S1 nDH data show distinct patterns, as compared to the L2L3, L3L4 and L4L5 discs, which all displayed similar nDH values across the disc area at the upright and flexed positions (Fig. 1). Discs from L2L3 to L4L5 have the smallest nDH at the posterior (≈0.5) and anterior (≈0.7) regions in the upright and flexed positions, respectively. L5S1 nDH at corresponding locations are much greater, with values of approximately 0.7 (p < 1e-04) and 1 (p < 1e-05), respectively (Fig. 2).

The L5S1 nDH is smallest (≈0.5–0.6) at the left and right regions of the discs in both upright and flexed positions (Fig. 2); these were significantly lower than the left (p < 0.01) and right (p < 1e-04) regions of the other discs (nDH ≈0.7–1.0). In general, nDH at the left and right regions of the disc becomes progressively smaller moving from the cranial to caudal intervertebral levels (Fig. 1a). This pattern appears consistent with disc height patterns measured in the supine and axially twisted positions^38^ and may be attributed to the increased inferior endplate concavity of lower lumbar vertebrae observed in previous lumbar morphometry studies^39,40^.

**Figure 3:**
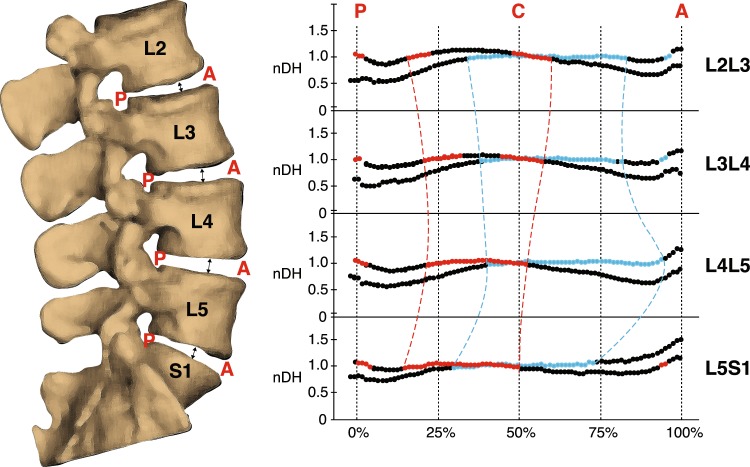
Average normalized disc height (nDH) along the AP axis of the disc at each segment level. Black-blue lines represent nDH at the upright position, while black-red lines represent nDH at the flexed position. The colored (blue or red) sections of each line indicate nDH values from 0.95–1.05 (±5% of central nDH) which roughly correspond to the nucleus pulposus of the disc. Superimposed colored and dashed lines illustrate differences in NP migration between segment levels.

The regions within the discs exhibiting nDH approximately equal to one (0.95–1.05) span approximately 50% to 66% of the disc width (medial-lateral axis) and 40% to 50% of the disc depth (anterior-posterior axis) in the upright position. These areas roughly correspond to the location of the hydrostatically pressurized and incompressible nucleus pulposus (NP) component of the discs^41,42^. At the flexed position, these regions are shifted posteriorly relative to their location in the upright position (Figs 1 and 3). Past *ex vivo* magnetic resonance imaging (MRI)-based studies have also reported NP posterior and anterior migration in the presence of joint flexion and extension, respectively^43,44^. While the current results reinforce this notion, subtle segment-specific differences are identified: distributions of nDH along the anterior-posterior axis of the L2L3 and L3L4 show similar trends at both positions; however, compared to the superior discs, the NP regions are more anterior in L4L5, and more posterior in L5S1 at the upright position (Fig. 3).

### Intervertebral disc strain

**Figure 4:**
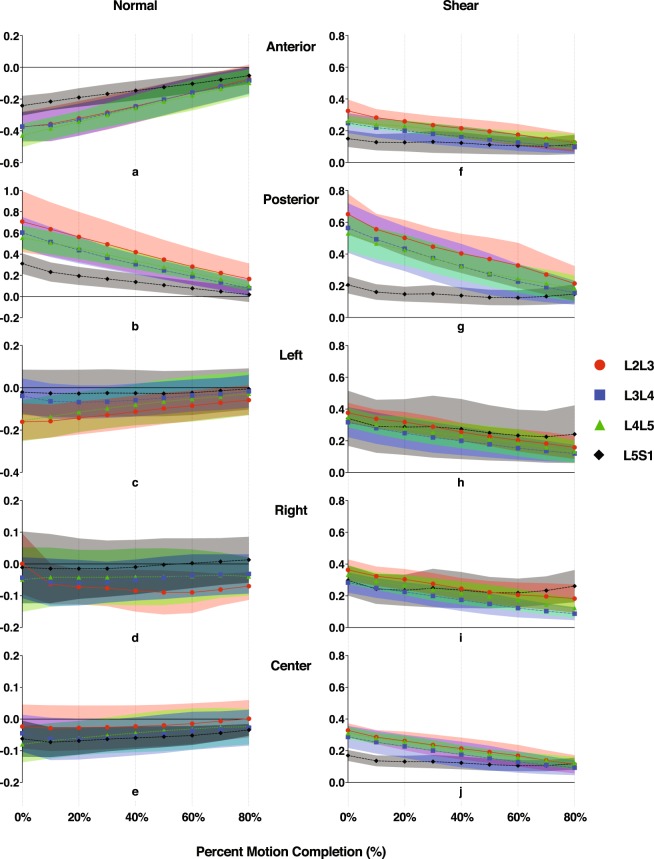
Level-specific disc strains at five regions of the discs throughout the lumbar extension motion. Normal (**a–e**) and shear (**f–j**) strains were computed at each decile of percent motion completion (%MC) from the flexed position (0%MC) to the nearly upright position (80%MC). Average magnitudes of the strains are presented. Color bands represent CI_95_ values, where non-overlapping bands indicate a significant pair-wise difference between discs at different segment levels. Note: Y-axis limits and scales of (**a–e**) vary depending on disc region.

External load magnitude variation administered in the current study appears to have little or no effect on the normal or shear strain at any of the five regions. Furthermore, Post-hoc Tukey results indicate no effect of external load magnitude on any regional normal or shear strain at the flexed position. Therefore, normal and shear strain data for trials of differing external load magnitudes are pooled before displaying the instantaneous strains over the entire ROM (Fig. 4).

Normal strains at the anterior and posterior regions demonstrate strong linear correlations with the amount of lumbar flexion, as indicated by high *R*^2^ values resulting from linear regressions with percent motion completion (%MC) as the single explanatory variable; correlations for normal strains at the left, right, and center are moderate or weak (Table 1, Fig. 4). Shear strains at all regions of the L2L3, L3L4, and L4L5 discs demonstrate strong linear correlations with lumbar flexion as well, while correlations at the L5S1 are notably weaker (Table 1, Fig. 4).

**Table 1:** Linearity coefficients of normal and shear strain vs. percent motion completion at five regions of the disc.

Disc	Normal strain					Shear strain				
	Anterior	Posterior	Left	Right	Center	Anterior	Posterior	Left	Right	Center
L2L3	0.92	0.97	0.43	0.54	0.44	0.84	0.86	0.83	0.86	0.86
L3L4	0.94	0.97	0.49	0.52	0.51	0.83	0.87	0.80	0.88	0.87
L4L5	0.97	0.99	0.78	0.38	0.69	0.81	0.84	0.88	0.79	0.85
L5S1	0.88	0.92	0.65	0.46	0.62	0.44	0.50	0.43	0.36	0.47

The linear R-squared coefficients were computed for each participant, segment, and disc region, and then were averaged across all participants.

**Figure 5:**
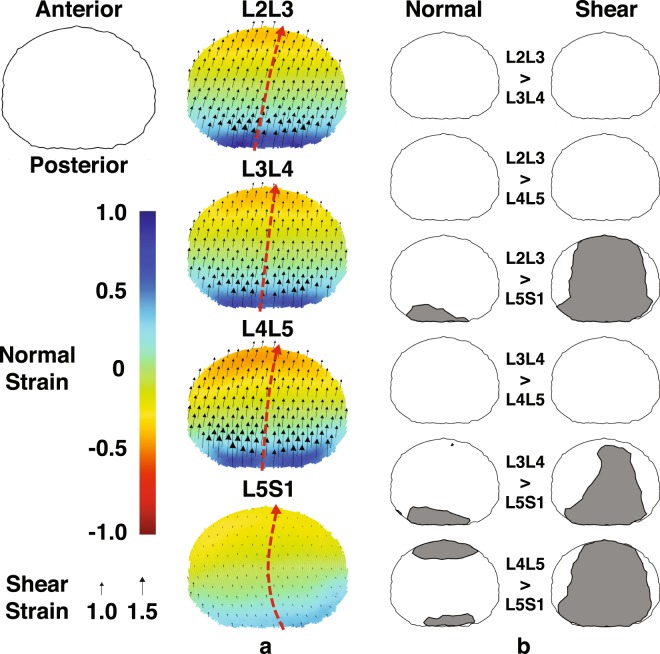
Mapping of disc strain across the axial planar surfaces of the lumbar discs from the L2 to S1 at the flexed position with respect to the upright reference frame. (**a**) Distribution of the average normal (color) and shear (black arrows) strains across the entire disc at the flexed position. Positive (more blue) and negative (more red) values indicate distraction and compression of the disc, respectively, while zero normal strain is displayed as green. Black arrows indicate both the magnitude and direction of shear strains. Superimposed red arrows intersecting the center of the disc indicate the approximate change in shear strain direction from the posterior to anterior end of the disc. (**b**) Areas exhibiting significantly different normal (left) and shear (right) strain values between segment levels at the flexed position based on non-overlapping ±95% confidence intervals.

**Figure 6:**
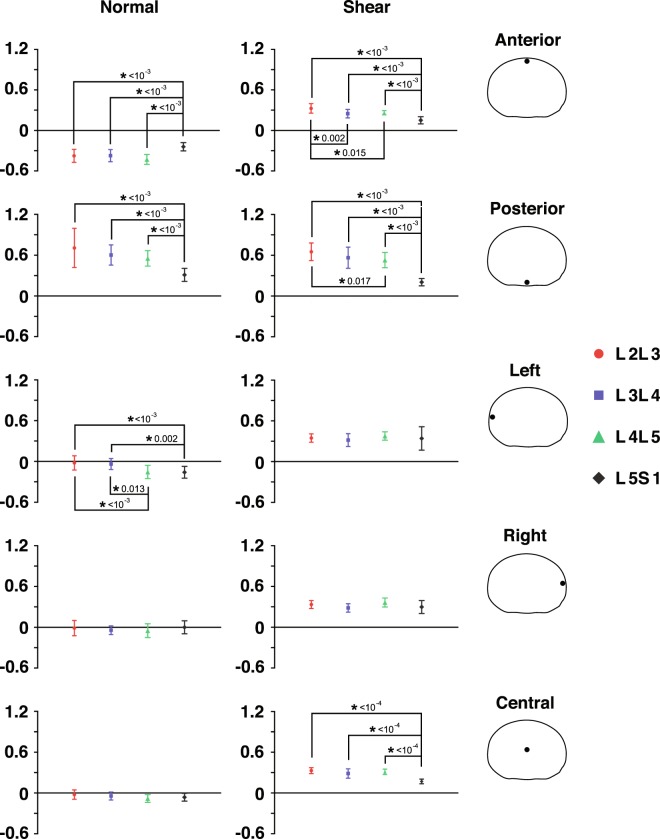
Average disc deformation of five regions of the disc at each segment level. Normal (left) and shear (right) deformations at the flexed position are computed at the anterior, posterior, left, right, and center regions of the disc. Error bars represent ±95% confidence intervals. An asterisk indicates a significant difference between segment levels and is accompanied by its associated p-value as assessed by Repeated Measures ANOVA.

The L5S1 disc displays unique shear strain patterns compared to the other discs. First, shear strain magnitudes (~0.2 on average) are significantly smaller across most of the disc cross-sectional area at the fully flexed position, as suggested by non-overlapping ±95% confidence intervals (Fig. 5b). Post-hoc Tukey tests (p < 0.001, Fig. 6) confirm this observation at the anterior and posterior regions. Second, L5S1 shear strains remain more or less constant over the entire ROM while the L2-L5 discs exhibit a linearly decreasing trend (Fig. 4f–j). This contrast is particularly noticeable in the posterior region of the discs, where shear strains in L2-L5 discs at the flexed position are significantly higher. Normal strain trends show similar differences: L5S1 exhibits significantly less distraction (p < 0.001) and compression (p < 0.001) compared to the other discs at the posterior and anterior regions, respectively (Fig. 6).

Further, L5S1 shear direction transitions gradually from about 120° to the medial-lateral (ML) axis in the posterior regions to about 80° in the anterior regions, indicating a changing anterior-posterior (AP) and ML coupled shear pattern from the posterior to the anterior region. On the other hand, the direction of shear remains more consistent throughout the other discs – approximately 75° to 85° off the ML direction (Fig. 5a).

Differences in L5S1 strain patterns compared to the other lumbar discs extend to the entire ROM. The clearest differences are seen in the posterior region, where L5S1 exhibits significantly smaller normal and shear strains through about half the range of motion (~50%MC). The anterior region shows a similar trend, although these are less pronounced than in the posterior region. For example, L5S1 anterior normal strain appears to be only significantly less than L4L5 (Fig. 4a), while L5S1 anterior shear strain is significantly less than the L2L3 and L4L5 from the flexed position through 20%MC, based on the CI_95_ values (Fig. 4f).

The center region of the L5S1 exhibits significantly less shear strain than all other discs (Fig. 6) at the flexed position (p < 1e-04) and at multiple time points during the lifting motion (Fig. 4j). No differences among segment levels are observed with regards to normal strain at the center of the disc. Interestingly, the left regions of the cranial levels (L2L3 and L3L4) exhibit significantly less normal strain than the caudal levels (L4L5 and L5S1) at the flexed position (L2L3: p < 0.001, L3L4: p < 0.02), while no differences in normal strain between segment levels are observed at the right region.

## Discussion

The data presented here demonstrate how dynamic X-ray imaging of the vertebral bone motion enables a detailed characterization and analysis of the morphometry and deformation of lumbar intervertebral discs *in vivo*. The results clearly show that the morphometry and deformation characteristics of the L5S1 disc are uniquely different from the rest of lumbar intervertebral discs. The substantial reduction of normal and shear strains at the L5S1 disc has three possible mechanistic explanations. First, the material properties of the L5S1 intervertebral disc’s individual components along with its morphological structure, together, yield a structure with greater stiffness compared to the cranial discs. While *in vivo* material property data for the discs remain unattainable, the effect of intervertebral disc height on segment stiffness determined by previous studies^45–47^ may suggest that the different disc height patterns observed at the L5S1 may play a role in facilitating increased segment stiffness, effectively reducing the magnitudes of normal and shear strain. Generally, these studies have found that a disc exhibiting lower disc height, typically measured at the center of the disc, would result in a stiffer motion segment of the spine. However, the effect of regional changes in disc height, or a significantly altered distribution in disc height, is not well understood. An alternative explanation is that the L5S1 disc simply experiences comparatively reduced loading. However, modeling studies have estimated L5S1 normal and shear loads to be comparable to discs at other levels or slightly larger^29,30^, implying a substantial disc load reduction being implausible. A third explanation is that the L5S1 disc, contrary to the other lumbar discs, is substantially more pre-loaded at the upright position compared to the flexed position. This would explain the smaller L5S1 strains observed throughout the lifting motion, as deformation of the disc at the upright position compared to its non-deformed state would remain undetected given that the upright position was used as the reference frame for computing disc deformation. Past studies have also observed significantly different behavior of the L5S1 when compared to other lumbar (L1-L5) segments, and have determined the L5S1 segment to have greater contribution during extension of the spine than in flexion^48,49^. Furthermore, disc degeneration and facet joint osteoarthritis have been found to occur independently at the L5S1, while associations between the two degenerative diseases were found at the L3L4 and L4L5^49^. These findings, along with the new insight from the current study, suggest that the mechanical environment of L5S1 and its related biochemical environment may be distinctly different from the other intervertebral discs.

Establishing deformation characteristics baselines in healthy lumbar intervertebral discs has important implications on the understanding and modeling of disc degeneration. Degenerative conditions in the intervertebral discs are often associated with changes in disc height and segment mobility, although the degree to which the *in vivo* mechanical environment causes these changes remains unclear. High mechanical strain of the disc tissues has been related to the secretion of inflammatory cytokines associated with disc degeneration and low back pain^19^. Therefore, knowledge of dynamic strain responses in the lumbar spine during a functional activity provides a crucial link between *in vivo* mechanical and biochemical milieus of the intervertebral discs in understanding different cellular responses *in vivo*.

It is envisioned that the data from the current study will add a critical piece of scientific evidence for designing treatments aimed at mitigating low back pain attributed to mechanically damaged or degenerated discs and restoring spine function. There has been much discussion surrounding the comparison of lumbar fusion – the current gold standard procedure – and various artificial disc replacement strategies as potential alternative surgical approaches for treating low back pain. Despite a theoretical mobility advantage offered by the total disc replacement, several clinical trials and meta-analyses failed to find sufficient evidence to support the claim^50^. The majority of current total disc replacement techniques focus on emulating the biomechanics of a spine motion segment as a whole but pay little attention to the mechano-physiological characteristics of the disc^51^. However, mimicking a healthy disc’s mechanical responses, i.e., motion and deformation, is the ultimate goal of implants designed to achieve full functional restoration^11^. To date, attempts to replicate the physiological elastic-type characteristics or the more ‘organic’ aspects of intervertebral discs have been unsuccessful^51^. Critically missing in the prior efforts are data and knowledge regarding *in vivo* loading and deformation behavior of the intervertebral discs^16^.

## Methods

### Experimental data acquisition

The original data were acquired from an experiment in which 14 healthy adults (8 male, 6 female; aged 19–30) participated and performed load-lifting tasks while their lumbar motions were recorded using a dynamic stereo-radiography (DSX) system^37,52^. The experimental protocol was approved by the Institutional Review Board (IRB) of the University of Pittsburgh, and was carried out in accordance with IRB ethical guidelines and regulations for conducting human subject studies. All participants provided written, informed consent prior to participating in the study. Briefly, participants lifted an object of varied weight (4.55 kg, 9.1 kg, or 13.6 kg), primarily using torso extension without bending of the knees. The bi-plane DSX system imaged the participants’ lumbar region, from anterior-posterior (AP) and medial-lateral (ML) directions, while they performed the lifting motions and when they assumed a static standing position (30 frames per second, 4 ms pulsed exposure). Three-dimensional (3D) lumbar vertebral kinematics were then determined via a volumetric model-based tracking algorithm which registered digitally reconstructed radiographs of CT-based models of each bone to radiographs recorded by the DSX system (accuracy ≤0.5°; 0.3 mm^53^). The analysis reported here includes data of 10 participants, as 4 other participants’ data were not consistently trackable due to inadequate image quality from data acquisition^52^.

### Disc height and deformation definitions

**Figure 7:**
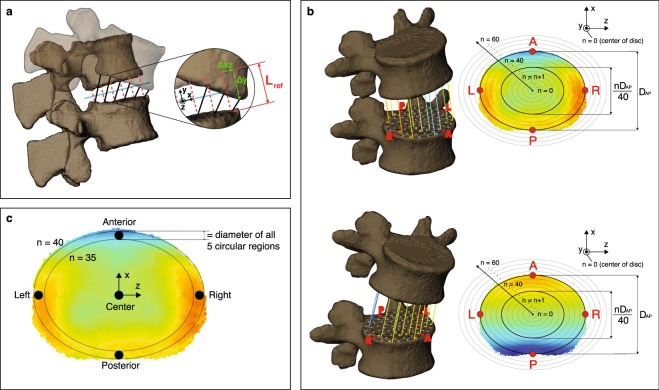
Mapping of disc morphometry and deformation of the intervertebral discs. (**a**) Schematic showing definitions of upright reference (L_ref_) and instantaneous disc height (black lines), as well as normal (Δy) and shear (Δxz) translations. (**b**) Schematic showing the process of mapping disc height (top) and deformation (bottom) of the line segments to a 2D elliptical grid of points across the whole disc. Shear strain is not shown for clarity purposes. The color of each line segment corresponds to a particular magnitude of disc height or deformation. (**c**) Definition of five circular regions of the disc for comparing level-specific disc morphometry and deformation (not shown) characteristics.

The CT-acquired vertebral bone models were sampled with a 0.8 mm spacing and imported into MATLAB (R2016b, Mathworks Inc., Natick, MA) as triangular meshed surfaces. With the 3D kinematics determined from the model-based tracking process, we placed the L2 to S1 vertebrae in their respective 3D orientation and position corresponding to the participant’s upright position. A computer algorithm was developed to automatically generate a representation of each intervertebral disc as approximately 4000 line segments (exact number varies by bone size) perpendicular to the transverse plane—defined as the average of planes fit to the adjacent endplates in the static upright position (Fig. 7a)^54^. Line segments extended from the centroid of each triangular element on the superior endplate surface of the inferior bone to the inferior endplate surface of the superior bone. Endpoints of the line segments remained connected to the endplates as the vertebrae moved relative to each other during lumbar motion. A characteristic ellipse was then fit to the superior endplate of the inferior vertebra, defined by the inferior endpoints of four line segments at the maximum anterior, posterior, left and right locations. The upright central disc height (h_c_) was defined as the length of the line segment nearest to the geometric center of the characteristic ellipse. At the upright and flexed positions, the instantaneous length of each line segment (h_i_) within each disc was normalized to the disc’s upright central disc height to obtain the upright and flexed nDH of all line segments forming the intervertebral disc ($nDH={h}_{i}/{h}_{c}$).

The intervertebral disc deformation is defined based on the relative motion between the two adjacent vertebral endplates, with the individual’s upright standing position as the reference (Fig. 7a). Nominal strains of the line segments were calculated with respect to the h_i_ values at the upright position – defined as L_ref_ – and were decomposed into two orthogonal components: normal strain, defined as $(\frac{\Delta y}{{L}_{ref}})$, and shear strain, defined as $(\frac{\Delta xz}{{L}_{ref}})$ (${L}_{ref},\Delta xz,\Delta y$ = upright disc height, displacement along average disc plane and normal displacement, respectively). By this definition, positive and negative values of normal strain corresponded to distraction and compression, respectively.

### Pointwise mapping of end-range disc height and deformation

In order to unify the nDH and strain values across all joint levels and participants, the geometry of each disc was re-sampled to consist of an identical number of line segments at the same locations relative to the disc’s size. First, the inferior disc surface was projected onto a 2D elliptical point grid consisting of 60 equidistant ellipses concentric to the disc’s characteristic ellipse and extending from the centroid up to 150% the size of the characteristic ellipse (Fig. 7b). The point grid extended 50% beyond the characteristic ellipse to ensure inclusion of the entire disc area, as the intervertebral disc is not perfectly elliptical. Second, sample points were evenly distributed along each elliptical profile, together forming a 2D point grid extending well beyond the outermost line segments of the disc’s cross-sectional area. The upright standing nDH at each point on the elliptical grid was then defined to equal that of the nearest original line segment (prior to re-sampling), resulting in a consistently sampled 2D plot of upright disc height over the entire disc area. Any point on the elliptical grid greater than 1 mm away from all line segments was considered to be outside of the disc region, and was therefore excluded from the 2D plot. By repeating this process at each intervertebral level across all participants, all discs were defined by approximately 8,000 distributed points scaled to their respective disc’s characteristic ellipse. At the flexed position, the same methodology was used to map the nDH and strain values (Fig. 7b) of all line segments to a 2D color and vector map.

### Computation of regional end-range disc height and deformation

The average nDH and deformation of the discs were quantified within five consistently identifiable regions: anterior, posterior, and central locations in the mid-sagittal plane; left and right locations in the mid-coronal plane. Each of the five regions was defined by a circular area on the superior endplate of the inferior vertebra, all with diameters equal to the AP distance between the 35^th^ and 40^th^ elliptical profiles (Fig. 7c). The average nDH and deformation among all line segments within each specified circular region were determined at the flexed and upright positions.

### Computation of instantaneous disc deformation

In addition to quantifying deformation at the flexed position, the average deformation of line segments within each of the five circular regions was tracked throughout the lumbar extension motion as well. Normal and shear strains were then plotted with respect to percent motion completion (%MC), a normalized representation of time based on the overall L2-S1 flexion angle, defined as ${\rm{ \% }}MC=(\frac{{\theta }_{c}-{\theta }_{i}}{{\theta }_{f}-{\theta }_{i}})\times 100{\rm{ \% }}$ (i, c, f = initial, current, and final L2-S1 lumbar flexion angle).

### Statistical analysis

Where data were successfully recorded from both trials per load for a participant, the two datasets were averaged into a single dataset to represent the participant’s motion for subsequent analyses. Level-specific differences in upright and flexed disc height were determined by identifying regions of the disc exhibiting location-specific differences in nDH. At each segment level, the mean and 95% confidence interval (CI_95_) of the mean nDH at the upright and flexed positions were calculated at every elliptical point corresponding to the same relative disc location. Each point exhibiting non-overlapping CI_95_ between segment levels or external load magnitudes indicated segment-wise or load-wise differences, respectively. Points close in proximity (within 3 mm) were grouped together to form anatomical areas of significantly different nDH characteristics. Any area containing less than three points was considered an outlier and was deemed insignificant. The same methodology used to quantify nDH differences was also utilized to determine areas of segment-wise or load-wise differences in normal and shear strain at the flexed position. As the reference frame for disc deformation was the upright standing position, deformation needed not be analyzed at this position as it was equal to zero.

Time series plots (“time”, as indicated by %MC progression) of the instantaneous normal and shear strains at five distinct circular regions defined above — the anterior, posterior, left, right, and center — of disc at each segment level were generated. CI_95_ of the mean normal and shear strain at every decile of %MC from 0% to 80%MC were calculated. Instances of non-overlapping confidence intervals indicated time intervals for which deformation trends between the corresponding segment levels were significantly different. Data beyond 80%MC was not included in the time series data as multiple subjects failed to reach 90%MC during the lifting motion.

Repeated measures analysis with data compiled as a mixed model was employed to identify segment-wise and load-wise differences in nDH and total disc strains at the five regions. The restricted maximum likelihood (REML) approach was used for the analysis. Segmental level (four levels: L2L3, L3L4, L4L5, L5S1) and load magnitude [three levels: 4.54 kg (10 lb), 9.1 kg (20 lb), 13.6 kg (30 lb)] were the two within-subject fixed-effect factors while “participant” was the random factor. The dataset comprised 10 groups (subjects) and a total of 116 observations. Starting with a null or empty model, the model was progressively updated by adding the fixed-effect factors, as below:

*Empty Model Formula:* ~*1* + *Random effect: Participant;*

*Update 1: Fixed effects:* ~*Segment_Level;*

*Update 2: Fixed Effects:* ~*Segment_Level* + *Load_Level;*

Whenever a main or interaction effect was deemed significant, post-hoc Tukey Honest Significant Difference (HSD) comparison-of-means tests would follow to determine differences between the levels. The above-mentioned steps were implemented separately for each response variable. All analyses were performed using R® Statistical Software^55^.
